# A Novel Hybrid Gradient-Based Optimizer and Grey Wolf Optimizer Feature Selection Method for Human Activity Recognition Using Smartphone Sensors

**DOI:** 10.3390/e23081065

**Published:** 2021-08-17

**Authors:** Ahmed Mohamed Helmi, Mohammed A. A. Al-qaness, Abdelghani Dahou, Robertas Damaševičius, Tomas Krilavičius, Mohamed Abd Elaziz

**Affiliations:** 1 Department of Computer and Systems Engineering, Faculty of Engineering, Zagazig University, Zagazig 44519, Egypt; amhm162@gmail.com; 2 Engineering and Information Technology College, Buraydah Private Colleges, Buraydah 51418, Saudi Arabia; 3 State Key Laboratory for Information Engineering in Surveying, Mapping and Remote Sensing, Wuhan University, Wuhan 430079, China; 4 LDDI Laboratory, Faculty of Science and Technology, University of Ahmed DRAIA, Adrar 01000, Algeria; dahou.abdghani@univ-adrar.edu.dz; 5 Department of Applied Informatics, Vytautas Magnus University, 44404 Kaunas, Lithuania; tomas.krilavicius@vdu.lt; 6 Department of Mathematics, Faculty of Science, Zagazig University, Zagazig 44519, Egypt; abd_el_aziz_m@yahoo.com

**Keywords:** human activity recognition, feature selection, gradient-based optimizer, grey wolf optimizer, metaheuristic

## Abstract

Human activity recognition (HAR) plays a vital role in different real-world applications such as in tracking elderly activities for elderly care services, in assisted living environments, smart home interactions, healthcare monitoring applications, electronic games, and various human–computer interaction (HCI) applications, and is an essential part of the Internet of Healthcare Things (IoHT) services. However, the high dimensionality of the collected data from these applications has the largest influence on the quality of the HAR model. Therefore, in this paper, we propose an efficient HAR system using a lightweight feature selection (FS) method to enhance the HAR classification process. The developed FS method, called GBOGWO, aims to improve the performance of the Gradient-based optimizer (GBO) algorithm by using the operators of the grey wolf optimizer (GWO). First, GBOGWO is used to select the appropriate features; then, the support vector machine (SVM) is used to classify the activities. To assess the performance of GBOGWO, extensive experiments using well-known UCI-HAR and WISDM datasets were conducted. Overall outcomes show that GBOGWO improved the classification accuracy with an average accuracy of 98%.

## 1. Introduction

The widespread use of mobile and smart devices has increased the demand for various smart home and Internet of Things (IoT) applications [1]. One of the most important applications is the Internet of Medical Things (IoMT) [2]/Internet of Healthcare Things (IoHT) [3], in which a real-time tracking, detection, and surveillance system is required for monitoring people’s daily activities for medical diagnostics, healthy lifestyle purposes or assisted living environments [4]. In many cases, a such system uses mobile device (such as a smartphone) sensor data [5]. To this end, human activity recognition (HAR) is a necessary application for IoHT, which plays an essential role in medical care applications [6].

In previous decades, different techniques have been used for HAR, such as computer vision methods [7,8,9] that use cameras to track human motion and actions, and wearable devices that should be carried by users, such as wearable sensors [10], smartwatches [11], and smartphones [12,13]. Additionally, there are other techniques, such as environment installed sensors [14], and WiFi signals, which include three techniques, namely received signal strength [15], channel state information [16], and WiFi radar (micro-Doppler radar) [17]. Each of these techniques has its advantages and disadvantages. For instance, computer vision methods need good light conditions, and they raise significant concerns in terms of people’s privacy [18]. Wireless methods do not require additional installation, but they are still in their early stage, and they require more and more improvements. Using carried sensors, such as smartphones, is preferred because virtually everyone uses smartphones today, so it is easy to collect data and to track different motions and activities.

With the developments in context-aware and machine learning techniques, researchers have applied different methods for HAR using data collected from smartphones. Smartphones have gained significant popularity for HAR due to three reasons. The first one is the ubiquitous nature of these small devices, which are used by almost everyone. The second reason is because of the reliability and efficiency of the procured data, and the third reason is that less restrictions can be considered in terms of privacy concerns compared to the case of computer vision methods. Therefore, in recent years, a number of studies have been proposed using different artificial intelligence (AI) techniques, such as [19,20,21].

In general, feature selection (FS) plays a vital role in improving classification accuracy and reducing computation costs. Nature-inspired algorithms such as ant colony optimization [22], particle swarm optimization [23], artificial bee colony [24], firefly algorithm [25], artificial ecosystem-based optimization [26], marine predators algorithm [27], Harris hawks optimizer [28], grey wolf optimizer [29], polar bear optimization [30] and red fox optimization [31], not to mention many others [32], are applicable and robust algorithms for finding a subset of prominent features while removing the non-informative features.

Especially in HAR, FS methods are popular techniques that help in obtaining high accuracy rates [33,34]. However, there are some limitations that can affect the performance of FS methods. For example, obtaining high accuracy rates can only be achieved with the correct features since some features do not provide improvements to the classification accuracy. In addition, FS methods are prone to a large number of features (i.e., high dimensionality), which can result in a high computational cost. Thus, to overcome these limitations and challenges, an efficient FS method should fulfill certain criteria such as being light and fast and able to extract relevant features, lower the feature space dimension, and reduce computation costs in terms of time and resources.

Hybrid algorithms are important for increasing the feature selection capability. Hybridization aims to benefit from each underlying optimization method to create a hybrid algorithm while minimizing any significant drawbacks. Such hybridization can often enhance the performance of various systems on complex tasks [35,36,37].

In our study, we propose a new FS method to improve the HAR system using the hybridization of two algorithms, namely the gradient-based optimizer (GBO) and grey wolf optimizer (GWO). The GBO is a novel metaheuristic (MH) algorithm proposed by Ahmadianfar et al. [38]. The GBO was inspired by gradient-based Newton’s model, which has two operators, namely the gradient search rule and local escape operator. Moreover, GBO uses a set of vectors for exploring the search space. To our knowledge, this is the first study to apply GBO for feature selection. Meanwhile, the GWO algorithm is a swarm intelligence and MH algorithm inspired by the hunting mechanisms and leadership hierarchies of grey wolves [39]. The GWO has four types of grey wolves, called alpha, beta, delta, and omega. These types are applied to emulate leadership hierarchies. Furthermore, GWO has three hunting steps, called searching, encircling, and attacking prey. In recent years, the GWO has been adopted to solve various optimization tasks, including feature selection [40,41,42].

### Contribution

The main contribution of the current study is to provide an efficient HAR system using smartphone sensors. The proposed system uses advanced AI techniques to overcome the complexity and limitations of traditional methods. We investigated the applications of MH optimization methods to select the best features that enhance the performance of the proposed HAR system. The GBO and GWO have proven their performance in the literature, but their individual applications suffer from certain limitations, such as being stuck at the local optima and the slow convergence. Thus, the combination of GBO and GWO provides a more robust method that balances between exploration and exploitation stages, in which the combined method will overcome the local optima problem. In addition, the proposed GBOGWO models the features as input for the well-known classifier, support vector machine (SVM), which is applied to classify human activities. Furthermore, extensive experimental evaluations have been carried out to evaluate the proposed HAR system performance using a public dataset, called UCI-HAR [43], and to verify its significant performance in extensive comparisons with existing HAR methods. We applied several performance measures, and we found that the proposed GBOGWO achieved better results when compared to several existing methods. Additionally, we also used the WISDM dataset to verify the performance of the GBOGWO method.

The rest of the current study is structured as follows. Related works are highlighted in Section 2. The preliminaries of the applied methods are described in Section 3. The proposed GBOGWO system is described in Section 4. Evaluation experiments are studied in Section 5. Finally, we conclude this study in Section 6.

## 2. Related Work

In this section, we only focus on the recent related works of HAR using smartphones. For other HAR techniques, the readers can refer to the survey studies [10,18,44].

Ronao and Cho [45] proposed a deep convolutional neural network (CNN) for tracking human activities using smartphone sensors. They used the UCI-HAR dataset [43], which was also used in this paper to test the performance of our proposed method. Their method achieved an average accuracy of 94.79%. Ahmed et al. [34] proposed a hybrid FS method to improve HAR using smartphones. They applied both wrapper and filter FS methods using a sequential floating forward search approach to extract features and then fed these features to the multiclass support vector machine classifier. The proposed approach showed robust performance and achieved significant classification results. Chen et al. [46] applied an ensemble extreme learning machine method for HAR using smartphone datasets. They applied Gaussian random projection to generate the input weights of the extreme learning machine, which improves the performance of the ensemble learning. Additionally, they tested the proposed method with two datasets, and they obtained high accuracy rates on both datasets. Wang et al. [21] proposed an HAR system using deep learning. They proposed an FS method using CNN to extract local features. After that, they employed several machine learning and deep learning classifiers to recognize several activities from two benchmark datasets. Zhang et al. [47] proposed an HAR model, called HMM-DNN, which uses a deep neural network to model the hidden Markov model (HMM). The main idea of this hybrid model is to enhance the performance of the HMM using DNN to be able to learn suitable features from the learning datasets and improve the classification process. Cao et al. [48] proposed a group-based context-aware HAR method, called GCHAR. They used a hierarchical group-based approach to enhance the classification accuracy and reduce errors. The GCHAR uses two hierarchical classification structures, inner and inter groups, that are used for detecting transitions through the group of the activities. Wang et al. [49] proposed an HAR model using a new feature selection method combining both wrapper and filter and wrapper methods. Moreover, they studied the use of different entrails’ sensors of smartphones and their impacts on HAR. Sansano et al. [50] compared several deep learning models, including CNN, long short-term memory (LSTM), bidirectional LSTM (biLSTM), deep belief networks (DBN), and gated recurrent unit networks (GRU), for human activity recognition using different benchmark datasets. They found that the CNN methods achieved the best results. Xia et al. [51] proposed a hybrid HAR model that combined both CNN and LSTM. The hybrid model aims to automatically extract features of the proposed activities and classify these activities using a set of a few parameters. They evaluated the proposed model using different datasets, including the UCI-HAR dataset, which achieved an average accuracy of 95.78%. Moreover, a few studies have used swarm intelligence in the HAR field. For example, Elsts et al. [52] proposed an efficient HAR system using the multi-objective particle swarm optimization algorithm (PSO). The PSO was applied to select the appropriate features, which also leads to reduce computation time. They used a random forest (RF) to classify several activities. The results confirmed that the PSO improved the classification accuracy and reduced the computational cost. Abdel-Basset et al. [6] proposed a new HAR system, called ST-DeepHAR, which uses an attention mechanism to improve long short-term memory (LSTM). Two public datasets were utilized to evaluate the performance of the ST-DeepHAR, which showed significant performance.

## 3. Material and Methods

In this section, we describe the datasets used in our experiments. Furthermore, we present the preliminaries of gradient-based optimization (GBO) and grey wolf optimization.

### 3.1. UCI-HAR Dataset

Anguita et al. [43] have published a public dataset for activities of daily living. Thirty participating subjects were asked to follow a protocol for performing 6 activities using a waist-mounted smartphone, namely walking (WK), walking upstairs (WU), walking downstairs (WD), sitting (ST), standing (SD), and lying down (LD). A sampling rate of 50 Hz was used to collect the tri-axial linear acceleration and angular velocity of the smartphone accelerometer and gyroscope sensors. Each participant performed a sequence of activities in order. Hence, the raw signals of all activities were registered in one text file per participant. Due to the low sampling rate and high amount of noise, collected signals were filtered using a low-pass filter with a corner frequency 20 Hz. Then, body acceleration was separated from the gravity acceleration component in order to better extract representative features. After that, additional time and frequency-domain signals were generated from the filtered body/gravity tri-axial signals such as jerk (or time derivative), signal magnitude using Euclidean norm, and fast Fourier transformation (FFT). A total of 17 signals were obtained per subject. Time-domain signals were segmented using fixed-width sliding windows of a length of 2.56 s with 50% overlapping, and an equivalent rate was applied to FFT signals. Thus, each window contained approximately 128 data points of activity; such a selected segmentation rate is supposed to meet the activities of normal people, as justified in [43]. After that, many useful functions were applied to filter and segment the signals in order to extract the features including the mean, standard deviation, signal magnitude area, entropy, energy, autoregressive coefficients and the angle between vectors. Now, each activity window is represented by a 561-length vector. Authors have also published separate files for training and testing featured data where 70% of the data samples were randomly selected for training and the remaining 30% were the independent set for testing. Thus, the number of examples per activity for the training and testing is indicated in Table 1. The percentage of each activity in this dataset refers to a more or less balanced dataset. Hence, it is relevant to design and test different classification and recognition HAR models from an applicability point of view.

### 3.2. Gradient-Based Optimization (GBO)

Within this section, we introduce the basic concept of a new metaheuristic technique named GBO. In general, GBO simulates the gradient-based Newton’s approach. The GBO depends on two operators to update the solutions, and each one of them has its own task. The first operator is the gradient search rule (GSR) which is used to improve the exploration, while the second operator is the local escaping operator (LEO), which is used to enhance the exploitation ability.

The first process in GBO is to construct a population *X* with *N* solutions, randomly generated using the following equation:

(1) $$x_{i}=x_{min}+rand\times \left(x_{max}-x_{min}\right),i=1,2,...,N$$

where $x_{min}$ and $x_{max}$ are the limits of the search space and rand∈[0,1] denotes a random number. Then, the fitness value for each solution is computed, and the best solution is determined.

Thereafter, the gradient search rule (GSR) and direction movement (DM) are applied to update the solutions ($x_{i}^{It},i=1,2,...,N$) in the direction ($x_{b}-x_{i}^{It}$) (where $x_{b}$ refers to the best solution). This updating process is achieved by computing new three solutions $x1_{i}^{It},x2_{i}^{It}$ and $x3_{i}^{It}$ as

(2) $$x1_{i}^{It}=x_{i}^{It}-GSR+rand\times \rho_{1}\times \left(x_{b}-x_{i}^{It}\right)$$

In Equation (2), $\rho_{1}$ is applied to improving the balance between exploitation and exploration during the optimization process and it is defined as

(3) $$\rho_{1}=2\times rand\times \alpha -\alpha$$

where:

α=|β×sin(3π/2+sin(β×3π/2))|

$$\beta =\beta_{min}+\left(\beta_{max}-\beta_{min}\right)\times {\left(1-{\left(It/Max_{It}\right)}^{3}\right)}^{2}$$

where $\beta_{min}=0.2$ and $\beta_{max}=1.2$. Iter denotes the current iterations, and $Max_{It}$ is the total number of iterations. The gradient search rule (GSR) is defined as follows:

(4) $$GSR=randn\times \rho_{2}\times \left(2\times \Delta x\times x_{i}^{It}\right)/\left(yp_{i}-yq_{i}+\epsilon \right)$$

with:

$$\Delta x=rand\left(1:N\right)\times |(\left(x_{b}-x_{r1}^{It}\right)+\delta )/2|$$

$$\delta =2\times rand\times (|\left(x_{r1}^{It}+x_{r2}^{It}+x_{r3}^{It}+x_{r4}^{It}\right)/4-x_{i}^{It}|)$$

where rand(1:N) is a random vector whose dimensions *N*, r1,r2,r3, and r4 refer to random integers selected from [1,N]. $\rho_{2}$ is formulated as defined by Equation (3).

The locations $yp_{i}$ and $yq_{i}$ are updated using Equations (5) and (6):

(5) $$yp_{i}=rand\times \left(x_{s}+x_{i}\right)/2+rand\times \Delta x$$

(6) $$yq_{i}=rand\times \left(x_{s}+x_{i}^{It}\right)/2-rand\times \Delta x$$

with:

(7) $$x_{s}=x_{i}^{It}-randn\times \rho_{1}\times \left(2\times \Delta x\times x_{i}^{It}\right)/\left(x_{b}-x_{worst}+\epsilon \right)$$

(8) $$x2_{i}^{It}=x_{b}-GSR+rand\times \rho_{2}\times \left(x_{r1}^{It}-x_{r2}^{It}\right)$$

(9) $$x3_{i}^{It}=x_{i}^{It}-\rho_{1}\times \left(x1_{i}^{It}-x2_{i}^{It}\right)$$

Finally, based on the positions $x1_{i}^{It}\;x2_{i}^{It}$, and $x3_{i}^{It}$, a new solution at iteration It+1 is obtained:

(10) $$x_{i}^{It+1}=r_{a}\times \left(r_{b}\times x1_{i}^{It}+\left(1-r_{b}\right)\times x2_{i}^{It}\right)+\left(1-r_{a}\right)\times x3_{i}^{It}$$

where $r_{a}$ and $r_{b}$ denote two random numbers.

Moreover, the local escaping operator (LEO) is applied to improve the exploitation ability of GBO. This is achieved by updating the solution $x_{i}^{It}$ using the following equation according to the probability pr:

(11) $$x_{i}^{It+1}=\left\{\begin{array}{cc} x_{i}^{It}+f_{1}\times W_{1}+f_{2}\times \rho_{1}\times W_{3}+u_{2}\times W_{2}/2 & pr<0.5\\ x_{b}+f_{1}\times W_{1}+f_{2}\times \rho_{1}\times W_{3}+u_{2}\times W_{2}/2 & otherwise \end{array}\right.$$

$$W_{1}=\left(u_{1}\times x_{b}-u_{2}\times x_{k}^{It}\right),$$

$$W_{2}=\left(x_{r1}^{It}-x_{r2}^{It}\right),$$

$$W_{3}=\left(u_{3}\times \left(x2_{i}^{It}-x1_{i}^{It}\right)\right)$$

In Equation (11), $f_{1}\in \left[-1,1\right]$ and $f_{2}$ denote a uniform random number and normal random number, respectively. $u_{1},u_{2}$, and $u_{3}$ are three random numbers defined as

(12a) $$\begin{array}{cc} \; & u_{1}=L_{1}\times 2\times rand+\left(1-L_{1}\right) \end{array}$$

(12b) $$\begin{array}{cc} \; & u_{2}=L_{1}\times rand+\left(1-L_{1}\right) \end{array}$$

(12c) $$\begin{array}{cc} \; & u_{3}=L_{1}\times rand+\left(1-L_{1}\right) \end{array}$$

where $L_{1}$ represents a binary variable (i.e., assigned to 0 or 1). Therefore, the new solution is obtained using the following equation:

(13) $$x_{k}^{It}=L_{2}\times x_{p}^{It}+\left(1-L_{2}\right)\times x_{rand}$$

where $L_{2}$ is similar to L−1 and $x_{p}^{It}$ refers to a selected solution from *X*, and $x_{rand}$ denotes a random solution obtained using Equation (1).

The main steps of the GBO algorithm are presented in Algorithm 1.
**Algorithm 1** The Gradient-Based Optimizer (GBO) 1:Initialize the parameters of GBO: ϵ,pr, $Max_{It}$ Maximum Iteration number, *N*: Population size. 2:Initialize randomly the population of N vectors using Equation (1) 3:Evaluate the position of each vector using the fitness function fit 4:Determine the best and worst solutions: $x_{best}$, $x_{worst}$ 5:Let It=1 6:**while**$It\leq Max_{It}$**do** 7: **for** each vector $x_{i}^{It}$ **do** 8:  Choose four integers randomly in the range [1..N] such that: r1≠r2≠r3≠r4 9:  Update the position of the vector $x_{i}^{It+1}$ using Equation (14).10:  Evaluate the quality of the vector $x_{i}^{It+1}$ using the fitness function $fit_{i}$11: **end for**12: **if** rand<pr **then**13:  Update the position of $x_{i}^{It+1}$ using the first branch of Equation (11)14: **else**15:  Update the position of $x_{i}^{It+1}$ using the second branch of Equation (11)16: **end if**17: Determine the best and worst solutions: $x_{best}$, $x_{worst}$18: It=It+119:**end while**20:Return the optimal solution $x_{best}$

### 3.3. Grey Wolf Optimization

In this section, the steps of the grey wolf optimization (GWO) [39] are described. The GWO emulates the behaviors of wolves in nature during the process of catching the prey $X_{b}$. The GWO has three groups of solutions named α,β, and γ—each of which has its own task and represents the first three best solutions, respectively, while the other solutions are called the μ group.

GWO starts by setting the initial value for a set of solutions *X* and evaluating the fitness value for each of them and determines $X_{\alpha }$, $X_{\beta }$, and $X_{\gamma }$. Thereafter, the solutions are updated using a set of approaches, such as the encircling technique, and it is formulated as [39]

(14) $$X^{t+1}=X_{pr}^{t}-B\times D,\;B=2b\times q_{1}-b,$$

(15) $$D=|A\times X_{pr}^{t}-X^{t}|,\;A=2q_{2}$$

where *A* and *B* denote the coefficient parameters, whereas $q_{1}$ and $q_{2}$ refer to random numbers generated from [0, 1]. The value of *b* sequentially decreases from 2 to 0 with an increase in the iterations as

(16) $$b=2-2\times t/t_{max}$$

where $t_{max}$ refers to the total number of iterations.

The second strategy in GWO is called hunting, and this solution can be updated using the following Equation [39]:

(17) $$X^{t+1}=\frac{\left(X_{1}+X_{2}+X_{3}\right)}{3},$$

(18) $$X_{1}=X_{\alpha }^{t}-B_{1}\times \left(D_{\alpha }\right),X_{2}=X_{\beta }^{t}-B_{2}\times \left(D_{\beta }\right)$$

$$X_{3}=X_{\gamma }^{t}-B_{3}\times \left(D_{\gamma }\right)$$

(19) $$D_{\alpha }=|A_{1}\times X_{\alpha }^{t}-X^{t}|,D_{\beta }=|A_{2}\times X_{\beta }^{t}-X^{t}|,D_{\gamma }=\left|A_{3}\times X_{\gamma }^{t}-X^{t}\right|$$

where $A_{k}=2q_{2},k=1,2,3$, and $B_{k}=2b\times q_{1}-b$.

The steps of GWO are listed in Algorithm 2 [39].
**Algorithm 2** Grey Wolf Optimization(GWO) 1:Initialize the population *X* of wolves $X_{i}\left(i=1,2,...,N\right)$ and parameters. 2:Find the fitness of the population. 3:Find alpha ($X_{\alpha })$, beta ($X_{\beta }$) and gamma ($X_{\gamma }$) solutions. 4:**while**$t<t_{max}$**do** 5: Update the position of each wolf $X_{i}\left(i=1,2,...,N\right)$ based on Equation (17). 6: Find the fitness of population. 7: Update alpha ($X_{\alpha }$), beta ($X_{\beta }$) and gamma ($X_{\gamma }$). 8: t=t+1 9:**end while**10:Return $X_{\alpha }$

## 4. Proposed Approach

**Figure 1:**
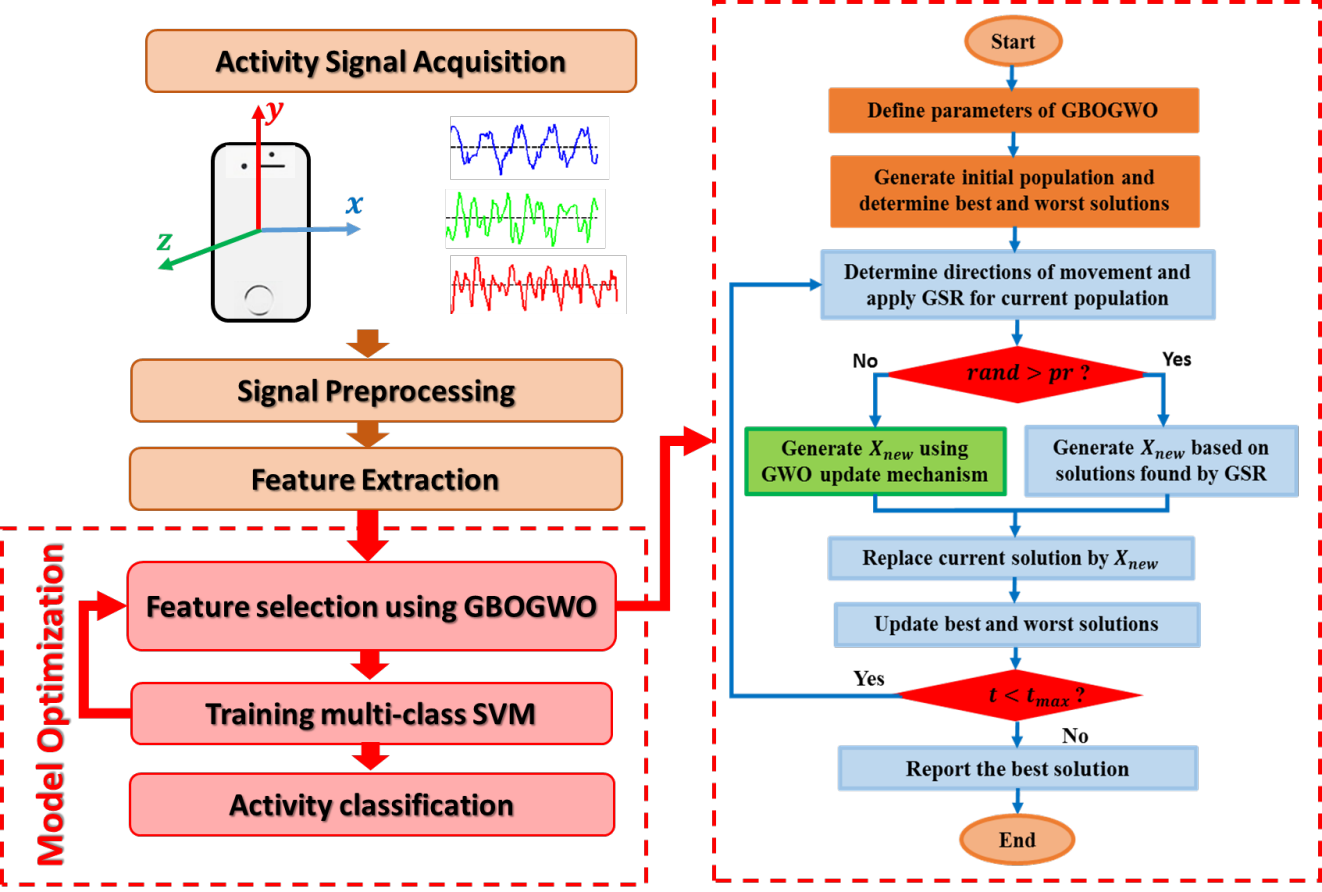
HAR model optimization framework (**left**) and GBOGWO flow chart (**right**).

Within this section, the steps of the developed HAR method based on a modified version of the GBO are introduced. The framework of the developed HAR method is given in Figure 1. The developed method starts by receiving the input data and splits them into the training and testing sets. This is followed by determining the initial value for the parameters of the developed HAR model such as the population size, the total number of generations, and the probability pr. Then, the initial population *X* is generated and the quality of each solution $X_{i},i=1,2,...,N$ is evaluated. This is achieved through two steps; the first step is to convert $X_{i}$ into a binary solution using the following equation:

(20) $$BX_{ij}=\left\{\begin{array}{cc} 1 & if\;X_{ij}>0.5\\ 0 & otherwise \end{array}\right.$$

where $BX_{i}$ is the binary form of $X_{i}$. The second step is to remove the features corresponding to zeros in BX, which represent irrelevant features. Then, those selected features from the training set are used to learn the multiclass-SVM classifier and compute the fitness value as [43,51]

(21) $$Fit_{i}=\lambda \times \eta_{i}+\left(1-\lambda \right)\times \left(\frac{|BX_{i}|}{Dim}\right),\;\eta_{i}=\left(1-PR\right)$$

where PR presents the classification precision.

The next step in the developed model is to find the best solution $X_{b}$ and the worst solution. Then, the solutions are updated according to $X_{b}$ and the operators of GBO and GWO. Here, GWO is applied to enhance the local escaping operator (LEO) according to the value of pr. In the case of pr greater than the random value, the operators of GBO are used to generate a new solution. Otherwise, the operators of GWO are used. By comparing the fitness value of the new obtained solution with the current solution $X_{i}$, we select the best of them and remove the worst one. The process of updating the solutions is ongoing until it reaches the stopping criteria. Thereafter, the testing set is reduced according to the features obtained by the best solution and the performance of the predicted activities is computed using different classification measures.

Equation (22) illustrates the time complexity analysis of the GBOGWO algorithm:

(22) $$\begin{array}{cc} T^{GBOGWO}= & \;T_{init.}+TN\left(T_{GSR}+\left(1-p\right)T_{Xnew}^{GWO}+pT_{Xnew}^{GBO}+T_{upd.}\right)\\ = & \;O\left(ND+NT_{FE}\right)+O\left(TND\right)+O\left(T_{FE}\right)\\ = & \;O(TND+NT_{FE}). \end{array}$$

where $T_{init.}$ represents the time spent collecting the initial population. *p* is the probability of selecting either the GWO update mechanism or GBO exploration subprocedure. $T_{GSR}^{GBO}$, $T_{Xnew}^{GBO}$ and $T_{Xnew}^{GWO}$ each has a time complexity of O(D). $T_{FE}$ refers to the time taken by the function evaluation, which has a notable enhancement in terms of execution time in HAR applications due to using classifiers such as multiclass-SVM, random forest, neural networks and others. $T_{upd.}$ denotes the time for evaluating $X_{new}$ and updating the best solution if necessary. *T* refers to the total number of iterations.

## 5. Experimental Results and Discussion

The proposed algorithm was applied to improve the classification performance of the UCI-HAR dataset via a feature selection approach. In this section, the experimental settings, the results of the proposed approach, the comparisons with other models, and the classification rates for the concerned dataset with comparison to other studies in the literature are presented. Moreover, a critical analysis of the obtained results using the proposed HAR system is given.

### 5.1. UCI-HAR Dataset

The performance of GBOGWO was exhaustively compared to a set of 11 optimization algorithms for feature selection. Basic continuous-based versions of the GBO, GWO, genetic algorithm (GA) [53], differential evolutionary algorithm (DE) [54], moth–flame optimization (MFO) [55], sine–cosine algorithm (SCA) [56], Harris hawks optimization (HHO) [57], and manta ray foraging (MRFO) [58] were implemented, in addition the particle swarm optimization (B-PSO) [59], bat algorithm (B-BAT) [60] and sine–cosine algorithm (B-SCA) [56]. The settings and parameter values of all algorithms used in the comparison are provided in Table 2.

As a classification task, true positive (TP), true negative (TN), false positive (FP) and false negative (FN) rates define the commonly used performance metrics for HAR systems, which are defined as follows:

(23) $$Accuracy=\frac{TP}{TP+TN+FP+FN}$$

(24) $$Precision\left(PR\right)=\frac{TP}{TP+FP}$$

(25) $$Recall/Sensitivity=\frac{TP}{TP+FN}$$

(26) $$Specificity=\frac{TN}{FP+TN}$$

Evaluation metrics of the comparison involve the mean (M) and standard deviation (std) of the precision (PR), M, and std of the number of selected features (# F), the percentage of feature reduction (red (%)), and the execution time. The Wilcoxon statistical test was used to determine the degree of significant difference between GBOGWO and each other compared algorithm in terms of the null hypothesis indicator *H* and significance level *p*-value. Each algorithm was repeated for 10 independent runs; this may be considered as the bottom line for examining the behavior of such a stochastic optimization technique. The reason refers to the huge execution time when training a multi-class SVM for extremely long training records (the training set was recorded with the dimension 561). The classification rates obtained by the proposed approach were compared to those of the original paper of the dataset under study as well as one recent study in the literature. Moreover, the performance of GBOGWO was compared to commonly used filter-based methods such as the *t*-test and *ReliefF* [61] in feature-selection applications.

All algorithms were implemented in the Matlab 2018a (MathWorks Inc., Natick, MA, USA) environment using CPU 2.6 GHz and RAM 10 GB.

### 5.2. Numerical Results of Experiments

**Figure 2:**
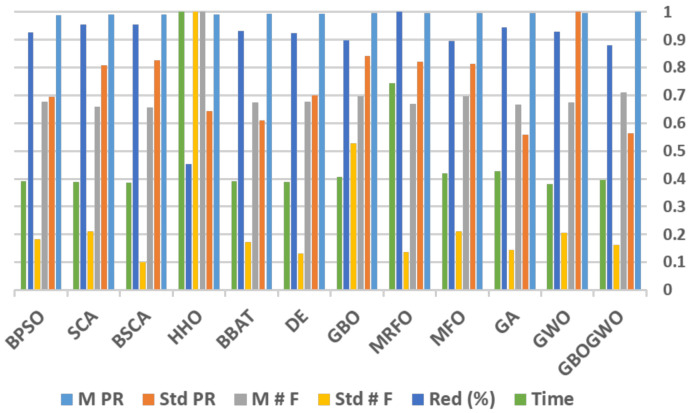
Normalized evaluation metrics of the compared algorithms.

Table 3 summarizes the results obtained for the proposed HAR model using various optimizers. The GBOGWO as a feature selector outperforms other techniques where the SVM model gives a PR of ≈98.13% using 304 features on average. Furthermore, average accuracy reaches 98%. Thus, the number of features is reduced from 561 to 304, which achieves a reduction ratio of 45.8%. The standard deviation of the proposed model, together with GA, are minimal (0.12 and 0.119, resp.) in this comparison. This reflects the good precision of the feature selection approach for this problem. However, the MRFO found a reduced feature set with a cardinality of 286.6 on average (i.e., a 52.12% reduction ratio), however, it seems that some important features were missing, thus the mean PR is 97.77%. Furthermore, HHO selected more features (approximately 428.6 on average), but the mean PR was only 97.25%. The results of the Wilcoxon test show that the performance of the GBOGWO is statistically distinguishable, where the *p*-value, which is <0.05 for all pairwise comparisons, together with H=1, reflects the superiority of the proposed technique. Under the experimental settings shown in Table 2, GBOGWO with the multiclass SVM model consumes 50.8 min on average for a single run. This execution time is very close to other faster optimizers such as GWO, BSCA, SCA, and DE with 49.02, 49.35, 49.8, and 49.8 min. In comparison, HHO takes a notable long execution time with 128.3 min. Figure 2 shows a summary of the reported results in Table 3 in a normalized fashion, which gives more clear intuition about the behavior of GBOGWO according to different evaluation metrics.

The confusion matrix, presented in Table 4, provides the rates of PR, sensitivity (Sens.), and specificity (Spec.) for each single activity. Walking downstairs (WD), lying down (LD), and walking (WK) were the highest recognized activities with PR rates of 100%, 100%, and 99.2%, respectively, while the worst PR rate was for standing (SD) activity with 93.57%. The recall of most activities was high except for sitting (ST) with 92.46%. It can also be noticed that the Spec. for all activities is quite good (>98.51%). The proposed model was able to well distinguish between the group of periodic activities (WK, WU, WD) and the other one of static or single-transition activities (ST, SD, LD) where the rate of misclassification is almost zero (only one wrong label between WU and ST in Table 4).

**Figure 3:**
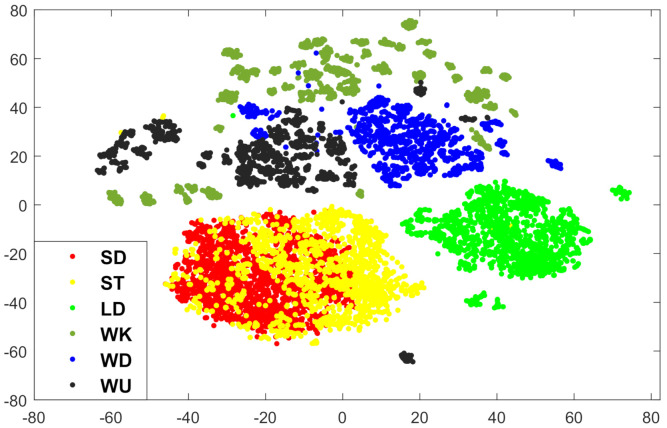
Original featured data [43] visualization in 2D.

Figure 3 presents 2D visualization for the basic feature records of activities (i.e., with 561 features) via carrying out principal component analysis and clustering. In Figure 3, (WK, WU, WD) in (dark green, blue, black) can be linearly separated from (SD, ST, LD) in (red, yellow, light green), except for very few records which are clustered to wrong classes between WU and ST. On the other hand, there is a high degree of similarity between the extracted features of each of SD and ST. Such similarity has complicated the classification task; thus, there is notable confusion between SD and ST (on average, 36 wrong labels in-between).

To summarize the conducted experiments, the proposed feature set for the UCI-HAR dataset in [43] was useful for the targeted recognition task; however, discarding some illusive features using the proposed technique proved very useful to improve the overall performance of such an HAR model. The feature set was successfully reduced by 45.8%, and at the same time, the mean PR reached 98.13%, and the mean accuracy was 98%.

### 5.3. Comparison with Other Studies

Recognition rates of the proposed HAR model were compared to each of the original studies of UCI-HAR dataset [43] and the recent study by [51]. In [43], 561D feature vectors were provided to a multiclass SVM, which gave a mean PR of 96%. A hybrid model using LSTM and CNN was applied to segmented sequences of activity signals in [51], which reported a mean PR of 95.8%. Table 5 shows a comparison of the results obtained herein and in the aforementioned studies. The notable improvement of whole model performance is noticed, in particular for WK and ST activities. However, the three models resulted in low precision for the SD activity.

### 5.4. Comparison with Filter-Based Methods

Filter-based methods such as the statistical tests and the Relief*F* algorithm [62] are commonly used for feature selection tasks. Such methods are time-efficient and their classifier-independent nature simplifies passing the selected feature set to any further classifier [63]. As a statistical test, the *t*-test examines the similarity between classes for each individual feature via mean and standard deviation calculations. It is then possible to rank features according to the significance importance and finally, define some cut-off threshold to select a feature set. The Relie*F* algorithm applies a penalty scheme, where features that map to different values for the same neighbors are penalized (i.e., negative weight); and otherwise rewarded. After that, the feature set with non-negative weights is expected to better represent the concerned classes.

Table 6 gives the results of the comparison between the proposed model and the filter-based approach using the *t*-test and Relief*F*. Relief*F* was able to extract the smallest feature set, achieving a reduction ratio of 67%, but the GBOGWO was outstanding, according to the resulting accuracy, sensitivity, and precision. However, the feature set selection using the *t*-test was enlarged to 350D, but this did not improve the performance. In Table 6, and for a typical value λ=0.99, the proposed GBOGWO fitness was 97.15%. For a more biased λ=0.9 towards reducing the feature set, the fitness of GBOGWO reaches 88.37%. For both cases of α, the proposed approach is superior to the examined filter-based methods.

The superior performance of the developed method over all other tested methods can be noticed from the previous discussion. However, the developed method still suffers from several limitations, such as the relatively large feature set required for achieving reasonable performance (i.e., 304 features on average for six activities). Thus, it is reasonable to realize such an HAR system on a smartphone environment to examine both the model size and real-time behavior. Moreover, enlarging the set of targeted activities is expected to add more time complexity for training a classifier such as the multi-class SVM.

### 5.5. Evaluate the Proposed GBOGWO with WISDM Dataset

For further evaluation, we test the proposed GBWGWO with other HAR datasets, called WISDM [64] dataset. This dataset contains six activities, namely walking (WK), walking upstairs (WU), walking downstairs (WD), sitting (ST), standing (SD), and jogging (JG). Table 7 shows the results of the proposed GBOGWO and several optimization methods, including the GWO, GA, MFO, MRFO, and GBO. From the table, we can see that the proposed method achieved the best results. It is worth mentioning that the best results for the WISDM dataset were achieved by using the random forest (RF) classifier; therefore, in this paper, for the WISDM dataset, we also used the RF.

**Figure 4:**
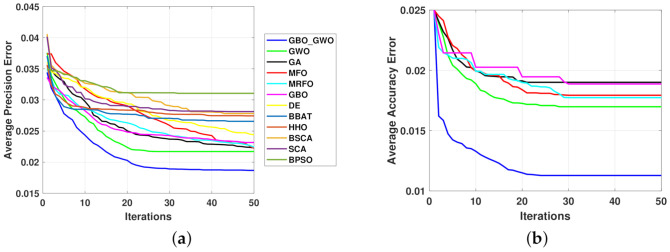
Convergence curves for the compared optimization algorithms: (**a**) UCI-HAR; and (**b**) WISDM.

A basic version of the RF algorithm with 50 decision trees gives an average accuracy of 97.5% for the feature set defined in Table 8. Following the pre-processing steps of the UCI-HAR dataset, each activity signal was separated into body acceleration and gravity component signals. Then, segments of a length of 128 points (i.e., same segment length used for UCI-HAR dataset) with 50% overlap were generated for the purposes of real-time applications. The feature set in Table 8 was generated using simple time-domain statistics in the three-axes of each segment, notably the mean, standard deviation (STD), the coefficients of the auto-regressive model (AR) in the order of 4, and the histogram counts where the number of bins is 5, among others. Moreover, the mean, max, and median frequencies of each segment in the three-axes enhance the feature set. Considering that the proposed features are generated for both the body signal and gravity component, then the cardinality of the feature set reaches 150. Thus, such a feature set can help distinguish the behavior of the compared algorithms for the WISDM dataset. Since previous studies that addressed the WISDM dataset have considered *Accuracy* to evaluate their algorithms, then the classification error is set to 1− mean(*Accuracy*) as shown in Figure 4b.

Since the search space of UCI-HAR—as a feature selection problem—is high-dimensional, then it is a suitable examiner for compared algorithms. Thus, for avoiding redundancy, only the top six algorithms according to the results in Table 3, namely GBOGWO, GWO, GA, MFO, MRFO, and GBO, were included in the experimentation of the WISDM dataset.

In Table 7, GBOGWO is able to achieve a mean accuracy (Acc) of 98.87%, which is a notable optimization for the basic model with a whole feature set of 97.5%. The GBOGWO outperforms other algorithms according to the Acc of classification only using 32.7 features on average (78.2% of reduction ratio). However, MFO uses the largest feature set among examined optimizers with 59.9 features, but it can reach a mean Acc of 98.21%. GBO attains the minimal feature set with cardinality of 25, but it seems insufficient to achieve a mean Acc above 98.11%. It was noticed that the STD for all algorithms was less than 0.01, which may refer to the relatively limited search space (e.g., the feature set size is 150). Moreover, the Wilcoxon test results in Table 7 ensure that GBOGWO is well distinguished from other algorithms of comparison.

In Table 9, the selection power of GBOGWO outperforms both the *t*-test and Relief*F* which tend to attain a large feature set of size 124 and 108, respectively, whilst lesser mean Acc of 97.58% and 98.11%, respectively. According to the fitness criteria defined in Equation (21), GBOGWO outperforms both methods in the case of giving most importance to Acc (i.e., λ=0.99) or to feature set reduction (i.e., λ=0.9).

Table 10 shows the confusion matrix of the test set, which represents 30% of whole samples. The activities ST, SD, and WK, were well recognized with the mean PR that exceeds 99.5%. It was noticed that the rates of PR, Sens. and Spec. were close for most activities which reflects that the classification model (features + classifier) was balanced between such metrics. Most conflicts occur between WU and WD, as well as between WU and JG where misclassifications reach 27 and 15, respectively. Such conflicts may be caused by the sensor position (in the pocket); thus, for such applications, it is suggested to collect activity signals from different positions on the body such as pocket, wrist, waist, and shoulder.

**Figure 5:**
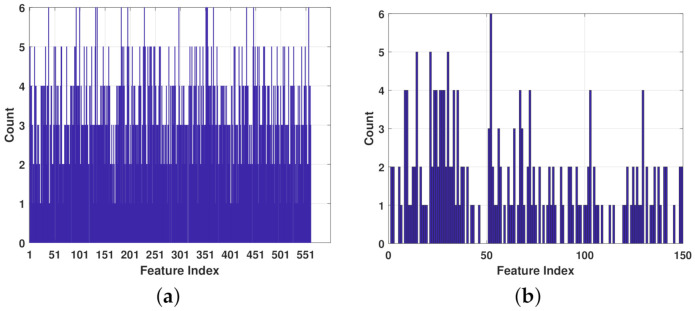
Feature frequency according to the top 6 algorithms for both datasets: (**a**) UCI-HAR; and (**b**) WISDM.

Figure 5 presents the selections of each of the top six algorithms for both datasets.

Table 11 focuses on the most frequent features in the optimized feature sets of each algorithm. For UCI-HAR, only features attained by all considered algorithms (e.g., count = 6) are shown. These features are generated from the body signals of both the accelerometer (BodyAcc) and gyroscope (BodyGyro) in both the time-domain (with the prefix *t*) and frequency-domain (with the prefix *f*). For more explanation of such features, the reader can refer to [43]. For WISDM, the skewness of the y axis of the body signal (Skewness-Y) looks like the most important feature as it is attained by every algorithm. Similarly, the tilt angle (TA), the STD of the jerk of x axis body signal (STD-Jerk-X), and the first coefficient of the AR model of magnitude signal (AR-Magnitude,1) have a frequency of 5. The maximum frequency of the z axis of the body signal (Max-Freq-Z) shows most notable effectiveness in the generated frequency-domain features with a count of 4. It is reasonable to find that body signal statistics are more useful than those of gravity components for such applications. Thus, only Gravity-STD-Y and Gravity-Kurtosis-Y appear in the elite feature set.

## 6. Conclusions and Future Work

In this study, we presented a robust human activity recognition (HAR) system based on data collected from smartphones. We developed a new feature selection (FS) method that was applied to enhance the HAR system using a hybrid MH algorithm that combine both gradient-based optimization (GBO) and grey wolf optimization (GWO). The proposed method, called GBOGWO, was applied to the SVM classifier to classify the activities of well-known UCI-HAR dataset. The combination of GBO and GWO overcomes the shortcomings of individual methods by exploiting the advantages of both algorithms to build an efficient FS method, which is employed to build a robust HAR classification system. Compared to existing HAR methods, and also to several metaheuristic algorithms that are applied as FS methods with SVM classifier, the developed GBOGWO has shown better performance in terms of classification accuracy and other performance metrics. Additionally, we evaluated the proposed GBOGWO with the WISDM dataset using the RF classifier. It also obtained the best results compared to several optimization algorithms.

The developed method could be further improved in future work to address more complex HAR datasets that may contain two or more human activities conducted simultaneously.

## Figures and Tables

**Table 1:** Summary of UCI-HAR activities data.

Activity	Abb.	Training	Test	Total	Per. (%)
Walking	WK	1226	496	1722	16.72
Walking upstairs	WU	1073	471	1544	14.99
Walking downstairs	WD	986	420	1406	13.65
Sitting	ST	1286	491	1777	17.25
Standing	SD	1374	532	1906	18.51
Lying	LY	1407	537	1944	18.11
Total		7352	2974	10299	100

**Table 2:** Search settings and parameter values of algorithms.

Search settings	lb	−10	Lower bound
	ub	10	Upper bound
	thr	0	Threshold
	*N*	30	Population size
	*T*	50	Maximum number of iterations
Algorithm	Parameter	Value	Description
GBOGWO	pr	0.75	Local escape probability
GBO	pr	0.5	Local escape probability
GA	*crossover*	0.4	Crossover percentage between individuals
	*mutation rate*	0.15	Rate of mutation operator
	*mutation per*	0.7	Percentage of mutation operator
MFO	*b*	1.5	Controlling shape of logarithmic spiral
SCA	*a*	2	Controlling range of sine and cosine waves.
B-SCA	*a*	2	Controlling range of sine and cosine waves.
	*TF*	V-shaped	$\left|\frac{2}{\pi }atan2\left(\frac{\pi }{2}\cdot x\right)\right|$
B-BAT	*A*	0.5	Loudness factor.
	*r*	0.5	Pulse rate
	$Q_{min}$	0	Minimum frequency
	$Q_{max}$	2	Maximum frequency
	*TF*	V-shaped	$\left|\frac{2}{\pi }atan2\left(\frac{\pi }{2}\cdot x\right)\right|$
B-PSO	$V_{max}$	0.5	Maximum velocity.
	$W_{max}$	0.9	Inertia value upper bound
	$W_{min}$	0.4	Inertia value lower bound
	$C_{1}$	2.78	Personal learning coefficient
	$C_{2}$	2.78	Global learning coefficient
	*TF*	S-shaped	$\frac{1}{1+\exp^{-x}}$

**Table 3:** Comparison of GBOGWO and other algorithms’ feature selection results.

	GBOGWO	GWO	GA	MFO	MRFO	GBO	DE	BBAT	HHO	BSCA	SCA	BPSO
M PR	98.13	97.82	97.8	97.77	97.73	97.68	97.55	97.34	97.25	97.21	97.18	96.89
Std PR	0.12	0.213	0.119	0.173	0.175	0.179	0.149	0.13	0.137	0.176	0.172	0.148
M # F	304	289.4	285	299.1	286.6	298.5	290.5	288.4	428.6	281.6	282.8	290.3
Std # F	11.09	14.16	9.82	14.51	9.29	36.15	8.92	11.87	68.56	6.85	14.38	12.47
Red (%)	45.8	48.41	49.19	46.68	52.12	46.79	48.12	48.59	23.6	49.8	49.73	48.25
*H*	-	1	1	1	1	1	1	1	1	1	1	1
*p*-value	-	$4.3\times 10^{-3}$	$2.4\times 10^{-4}$	$2.1\times 10^{-4}$	$2.3\times 10^{-4}$	$5.2\times 10^{-5}$	$1.3\times 10^{-5}$	$1.3\times 10^{-7}$	$3.2\times 10^{-8}$	$4.9\times 10^{-8}$	$1.7\times 10^{-7}$	$4.3\times 10^{-9}$
Time (min)	50.8	49.02	54.78	53.83	95.4	52.3	49.8	50.1	128.3	49.35	49.8	50.03

**Table 4:** Classification rates and confusion matrix of UCI-HAR activities using the proposed model.

		True Class							
	Activities	WK	WU	WD	ST	SD	LD	Sens.	Spec.
Predicted Class	WK	496	0	0	0	0	0	100	99.84
	WU	0	471	0	0	0	0	100	99.56
	WD	4	10	406	0	0	0	96.6	100
	ST	0	1	0	454	36	0	92.46	99.67
	SD	0	0	0	8	524	0	98.5	98.51
	LD	0	0	0	0	0	537	100	100
Precision (PR)		99.2	97.72	100	98.27	93.57	100	98.13	

**Table 5:** Comparison with other studies in the literature.

Ref.	Technique	Activities						PR (%)
		WK	WU	WD	ST	SD	LD	
[43]	All features+SVM	96	98	99	97	90	100	96
[51]	LSTM-CNN	94.65	95.03	100	92.32	93.61	100	95.8
ST-DeepHAR [6]	LSTM	99	96	99	95	99	98	97.66
Proposed technique	GBOGWO+SVM	99.2	97.72	100	98.27	93.57	100	98.13

**Table 6:** Comparison with filter-based methods.

Method	Classification Rates (%)			# F	Red (%)	Fitness Using Equation (21)	
	Acc.	Sens.	PR			λ=0.99	λ=0.9
*t*-test	95.12	95.15	95.53	350	37.6	94.58	86.04
Relief*F*	96.71	96.62	96.78	183	67	95.81	87.13
Proposed model	98	97.92	98.13	304	45.8	97.15	88.37

**Table 7:** Numerical results for WISDM.

	GBOGWO	GWO	GA	MFO	MRFO	GBO
M Acc	98.87	98.30	98.1	98.21	98.23	98.11
Std PR	0.006	0.002	0.01	0.009	0.013	0.019
M # F	32.7	27.9	43.5	59.9	29.7	25
Std # F	3.77	4.38	4.67	6.33	8.35	0.9
Red (%)	78.2	81.4	71	60	80.2	83.33
*H*	-	1	1	1	1	1
*p*-value	-	$6.5\times 10^{-9}$	$7.4\times 10^{-13}$	$1.7\times 10^{-13}$	$1.3\times 10^{-13}$	$2.6\times 10^{-13}$
Time (min)	65.67	58.57	89.88	67.28	117.28	78.13

**Table 8:** Feature engineering for the WISDM dataset.

Signals	Body Acceleration and Gravity Component
Time-domain *	AR coeff. (12); AR coeff. of magnitude (4); acceleration (1); entropy of jerk (3); histogram (15); kurtosis (3); mean (3); mean of jerk (3); mean of magnitude (1); max (3); mean of absolute difference (3); power of gravity (3); skewness (3); STD (3); STD of jerk (3); STD of magnitude (1); SMA (1); TA (1)
Frequency-domain	Mean freq. (3); max freq. (3); median freq. (3)
Cardinality	150

* AR coeff.: auto-regressive model coefficients; STD: standard deviation; SMA: signal magnitude area; TA: tilt angle. The enhancement of each statistic/coefficient is mentioned in parentheses.

**Table 9:** Comparison with filter-based methods for the WISDM dataset.

Method	Classification Rates (%)			# F	Red (%)	Fitness Using Equation (21)	
	Acc.	Sens.	PR			λ=0.99	λ=0.9
*t*-test	97.58	96.01	96.37	124	17.33	96.61	87.9
Relief*F*	98.11	96.85	97.32	108	28	97.14	88.37
Proposed model	98.87	98.02	98.54	32.7	78.2	97.88	89

**Table 10:** Classification rates and confusion matrix of WISDM activities using the proposed model. Test set represents 30% of total samples.

		True Class							
	Activities	WD	ST	SD	WU	WK	JG	Sens.	Spec.
Predicted Class	WD	426	0	0	27	0	2	93.63	99.85
	ST	0	266	1	3	0	0	98.52	100
	SD	0	0	216	0	0	0	100	99.98
	WU	7	0	0	539	0	15	96.08	99.28
	WK	0	0	0	0	1943	0	100	100
	JG	0	0	0	2	0	1588	99.87	99.51
Precision (PR)		98.38	100	99.54	94.40	100	98.94	98.54	

**Table 11:** Features selected by top 6 algorithms.

UCI-HAR (Count = 6)		WISDM		
Index	Name	Index	Name	Count
38	tBodyAcc-correlation()-X,Y	52	Skewness-Y	6
93	tBodyAccJerk-min()-X	14	TA	5
100	tBodyAccJerk-iqr()-X	21	STD-Jerk-X	5
132	tBodyGyro-max()-Z	30	AR-Magnitude,1	5
135	tBodyGyro-min()-Z	8	AR-Y,4	4
183	tBodyGyroJerk-entropy()-X	9	AR-Z,1	4
196	tBodyGyroJerk-arCoeff()-Z,3	23	STD-Jerk-Z	4
229	tBodyAccJerkMag-mad()	24	Mean-X	4
298	fBodyAcc-kurtosis()-X	26	Mean-Z	4
352	fBodyAccJerk-mad()-Y	27	STD-X	4
353	fBodyAccJerk-mad()-Z	28	STD-Y	4
355	fBodyAccJerk-max()-Y	33	AR-Magnitude,4	4
367	fBodyAccJerk-entropy()-X	35	STD-Magnitude	4
433	fBodyGyro-max()-X	67	Max-Freq.-Z	4
447	fBodyGyro-entropy()-Y	72	Acceleration	4
557	angle(tBodyGyroMean,gravityMean)	103	Gravity-STD-Y	4
		130	Gravity-Kurtosis-Y	4

## Data Availability

The UCI-HAR datasets used in this study are public datasets.
